# Insights into the evolutionary origin of the pineal color discrimination mechanism from the river lamprey

**DOI:** 10.1186/s12915-021-01121-1

**Published:** 2021-09-16

**Authors:** Seiji Wada, Emi Kawano-Yamashita, Tomohiro Sugihara, Satoshi Tamotsu, Mitsumasa Koyanagi, Akihisa Terakita

**Affiliations:** 1 Department of Biology and Geosciences, Graduate School of Science, Osaka City University, Osaka, 558-8585 Japan; 2 The OCU Advanced Research Institute for Natural Science and Technology, Osaka City University, Osaka, 558-8585 Japan; 3 Department of Chemistry, Biology and Environmental Science, Faculty of Science, Nara Women’s University, Nara, 630-8506 Japan

**Keywords:** Opsin, Pineal organ, Color opponency, Lamprey

## Abstract

**Background:**

Pineal-related organs in cyclostomes, teleosts, amphibians, and reptiles exhibit color opponency, generating antagonistic neural responses to different wavelengths of light and thereby sensory information about its “color”. Our previous studies suggested that in zebrafish and iguana pineal-related organs, a single photoreceptor cell expressing both UV-sensitive parapinopsin and green-sensitive parietopsin generates color opponency in a “one-cell system.” However, it remains unknown to what degree these opsins and the single cell-based mechanism in the pineal color opponency are conserved throughout non-mammalian vertebrates.

**Results:**

We found that in the lamprey pineal organ, the two opsins are conserved but that, in contrast to the situation in other vertebrate pineal-related organs, they are expressed in separate photoreceptor cells. Intracellular electrophysiological recordings demonstrated that the parietopsin-expressing photoreceptor cells with Go-type G protein evoke a depolarizing response to visible light. Additionally, spectroscopic analyses revealed that parietopsin with 11*-cis* 3-dehydroretinal has an absorption maximum at ~570 nm, which is in approximate agreement with the wavelength (~560 nm) that produces the maximum rate of neural firing in pineal ganglion cells exposed to visible light. The vesicular glutamate transporter is localized at both the parietopsin- and parapinopsin-expressing photoreceptor terminals, suggesting that both types of photoreceptor cells use glutamate as a transmitter. Retrograde tracing of the pineal ganglion cells revealed that the terminal of the parietopsin-expressing cells is located close enough to form a neural connection with the ganglion cells, which is similar to our previous observation for the parapinopsin-expressing photoreceptor cells and the ganglion cells. In sum, our observations point to a “two-cell system” in which parietopsin and parapinopsin, expressed separately in two different types of photoreceptor cells,  contribute to the generation of color opponency in the pineal ganglion cells.

**Conclusion:**

Our results indicate that the jawless vertebrate, lamprey, employs a system for color opponency that differes from that described previously in jawed vertebrates. From a physiological viewpoint, we propose an evolutionary insight, the emergence of pineal “one-cell system” from the ancestral “multiple (two)-cell system,” showing the opposite evolutionary direction to that of the ocular color opponency.

**Supplementary Information:**

The online version contains supplementary material available at 10.1186/s12915-021-01121-1.

## Background

Most non-mammalian vertebrates capture light by extraocular photoreception and utilize the light information for non-visual functions [1, 2]. Pineal-related organs are the most highly developed extraocular photoreceptive organs in non-mammalian vertebrates. Pineal-related organs in vertebrates from cyclostomes to reptiles have an ability to discriminate the wavelengths of light. This ability is based on “color” opponency, that is, an antagonistic light response to different wavelengths of light, e.g., UV and visible light [3–6].

We previously reported that parapinopsin, a UV-sensitive opsin first identified in the catfish [7], serves as a UV sensor in pineal color opponency systems [8–10]. In addition, we identified parapinopsin in several animals, in which pineal organs are reported to exhibit color opponency [11]. Therefore, parapinopsin is considered the common molecular basis of UV reception in the color opponency of pineal organs. In the zebrafish pineal organ, parapinopsin contributes to generating color opponency in the photoreceptor cells by serving as both UV and visible light sensors in a single photoreceptor cell because of its bistable nature, i.e., an interconvertibility between the UV-sensitive dark state and visible light-sensitive photoproduct by UV and visible light absorption, respectively [12]. Under natural “white” light composed of spectra distributed in UV and visible regions, parapinopsin forms a photoequilibrium-like state between the dark state (UV-sensitive) and photoproduct (visible light-sensitive), serving as sensors for two colors of lights. Therefore, the generation of color opponency with parapinopsin alone in a single photoreceptor cell requires high-intensity light (i.e., daytime) to form the photoequilibrium-like state between two states of parapinopsin.

In zebrafish, the color opponency-generating photoreceptor cells also contain green-sensitive parietopsin, which was first found in the side-blotched lizard parietal eye [13]. In a previous study, we also reported the co-expression of UV-sensitive parapinopsin and green light-sensitive parietopsin within the photoreceptor cells in the iguana parietal eye [10]. Although parapinopsin can generate color opponency without additional opsins under high-intensity light, parapinopsin-expressing cells also contain green-sensitive parietopsin. Based on previous findings on color opponency in the parietal eye of the side-blotched lizard [13], it can be speculated that parapinopsin and parietopsin together contribute to the generation of color opponency under “weak” light conditions (e.g., dawn and dusk). In the lizard parietal eye, it has been reported that a single photoreceptor cell expresses blue-sensitive pinopsin as well as green-sensitive parietopsin and the two opsins are responsible for generating color opponency against blue and green light via antagonistic phototransduction [13, 14]. Based on these findings, it is possible to speculate that parietopsin may be a common molecular basis for visible light reception in single photoreceptor systems of pineal wavelength discrimination.

Thus far, only single photoreceptor cell-based systems (“one-cell systems”) have been found in limited vertebrates, teleosts, and lizards [10, 12–14] as a cellular basis underlying pineal color opponency. The clear description about the molecular and cellular bases underlying pineal color opponency in the other non-mammalian vertebrates has not been given. To gain a deeper understanding about common mechanisms and evolution of the pineal color opponency, it is presumably important to address its molecular and cellular bases in “primitive” vertebrates, such as lampreys, because they are considered to inherit the ancestral vertebrate phenotypes. In the lamprey pineal organ, several ganglion cells show chromatic responses. It was reported that the maximum sensitivities in the inhibitory and excitatory responses are within the UV and green light regions [4]. We also identified parapinopsin as the UV-sensitive opsin responsible for the UV sensitivity in lamprey pineal color opponency [11]. However, the opsin responsible for the visible light sensitivity has not been identified. Therefore, we tested if parietopsin is involved in the lamprey pineal organ. Here, we found that parietopsin serves as a green-sensitive opsin also in the lamprey pineal organ, but it is localized in separate photoreceptor cells from the parapinopsin-expressing cells in this system.

## Results

### Identification of an opsin underlying visible light sensitivity in pineal color opponency

First, we investigated whether the river lamprey possesses a parietopsin gene. A partial DNA sequence coding an opsin-based pigment similar to pouched lamprey parietopsin [15] was found in the marine lamprey genomic sequence. With reference to its DNA sequence, we successfully isolated full-length cDNA from the river lamprey. The molecular phylogenetic tree of vertebrate visual and non-visual opsins, including parietopsin, shows that the isolated opsin falls into the parietopsin group, indicating that a parietopsin gene is present in the river lamprey (Additional file 1: Figure S1).

**Fig. 1:**
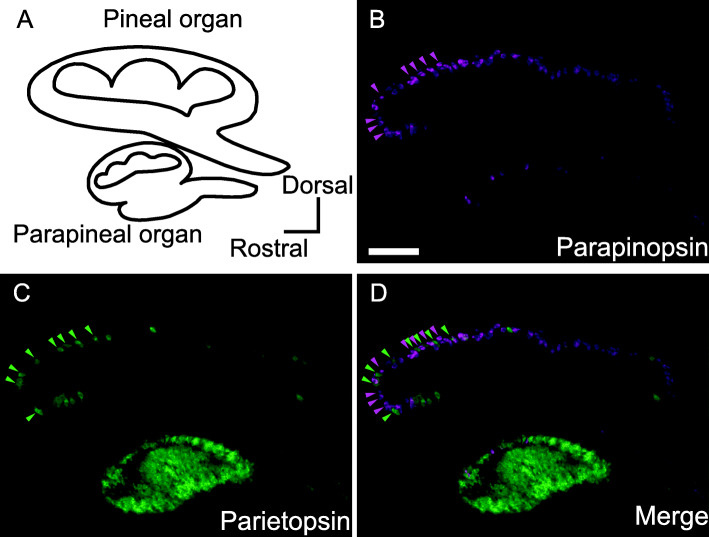
Expression patterns of parapinopsin and parietopsin in the lamprey pineal and parapineal organs. **A** Lamprey pineal and parapineal organ schematic. **B**–**D** Fluorescence images by double fluorescence in situ hybridization analyses showing expression patterns of parapinopsin (**B**, magenta) and parietopsin (**C**, green). The merged image shows parapinopsin and parietopsin express in separate cells (**D**). Scale bar = 100 μm

Next, we compared the expression patterns of parietopsin and parapinopsin. Double fluorescence in situ hybridization analysis revealed that parapinopsin and parietopsin are localized in separate cells and are not co-expressed (Fig. 1A–D, Additional file 1: Figure S2C and D). We also found that the distribution of the parietopsin-positive photoreceptor cells in the pineal organ is not identical to that of the parapinopsin-positive photoreceptor cells. The parapinopsin-positive cells are found in the dorsal and peripheral regions (Fig. 1B, Additional file 1: Figure S2A), which is consistent with our previous report [16]. In contrast, the number of parietopsin-expressing cells is larger in the periphery and its surrounding regions than in the central part of the dorsal region (Fig. 1C, Additional file 1: Figure S2B). The distributions of the two opsins share in the peripheral and its surrounding regions, where the ganglion cells showing “chromatic” property against UV and visible light are abundantly present [4].

The separate localization of these opsins is a different observation from those in iguana parietal eyes and zebrafish pineal organs. Additionally, we found that parietopsin is highly expressed in the parapineal organ, which is evolutionarily closely related to the lizard parietal eye, which expresses parietopsin.

**Fig. 2:**
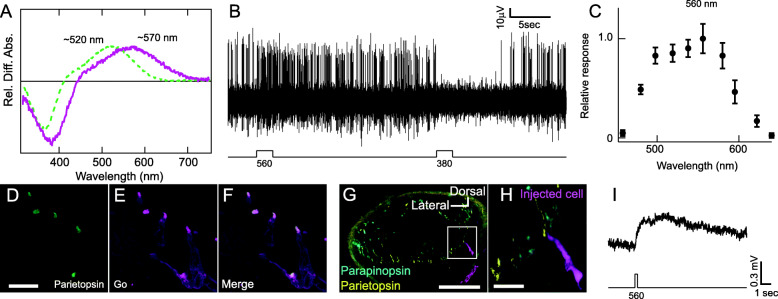
Parietopsin acts as the visible light sensor contributing to pineal color discrimination. **A** Relative difference absorption spectra of parietopsin reconstituted with 11*-cis* A1 (green, broken curve) and A2 (magenta, solid curve) retinal before and after light irradiation. Parietopsin with A1 retinal is a green light-sensitive pigment (*λmax*, ~520 nm) similar to previous reports. Reconstitution with A2 retinal shifts the parietopsin spectrum to longer wavelengths (*λmax*, ~570 nm). **B** Chromatic response recorded from pineal ganglion cells showing promotion and inhibition of neural firings to visible light (560 nm) and UV light (380 nm), respectively. **C** The relative response curve of the visible light-sensitive component (promotion of neural firings) in pineal chromatic responses, showing the response peak at 560 nm, consistent with the previous report [4] (*n* = 6 measurements, see the “Methods” section and Fig. S3 for the details). Error bars indicate SEM. **D**–**F** Fluorescence images by immunohistochemical analyses, showing parietopsin (**D**, green) and Go-type G protein (**E**, magenta) colocalized in the outer segment (**F**, merge). Scale bar = 20 μm. **G**–**I** Analysis of the photoresponse in a parietopsin-expressing photoreceptor cell by intracellular recording. **H** is the enlarged image from the white square in **G**. Note that the location of the pineal slice is shown in Fig. S2D. The cell dye loaded by neurobiotin injection (**G** and **H**, magenta) expresses parietopsin (yellow) but not parapinopsin (cyan), showing that the intracellular recording was from a single parietopsin-expressing cell. Note that the dye was loaded into the cell body but not into the parietopsin-containing outer segment because the outer segment consists of stacked membranes and limited cytosolic portion like the case of other photoreceptor cells (see Additional file 2: Movie S1). Scale bar = 100 μm (**G**), 20 μm (**H**). Intracellular recording analysis of a parietopsin-expressing photoreceptor cell shows a depolarizing response to visible light (**I**)

We next investigated the absorption spectrum of parietopsin from parietopsin-expressing cultured cells after adding 11-*cis* retinal (A1 retinal). The difference absorption spectrum obtained by subtracting the absorption spectrum after green light irradiation from that before the irradiation showed that lamprey parietopsin reconstituted with A1 retinal is a green-sensitive pigment with an absorption maximum at ~520 nm (Fig. 2A, green broken curve), similar to that of lizards [13, 17]. Because 11*-cis* 3-dehydroretinal (A2 retinal) is the major chromophore in the adult lamprey pineal organ [18], we reconstituted lamprey parietopsin with A2 retinal. The difference absorption spectrum of parietopsin with A2 retinal showed that the maximum absorption wavelength is ~570 nm (Fig. 2A, magenta solid curve). Electrophysiological analysis with the extracellular recording of the lamprey pineal organ revealed that the neural firings of the ganglion cells are excited and inhibited by visible (~560 nm) and UV (~380 nm) light irradiation, respectively, showing the chromatic response of the ganglion cell (Fig. 2B). We confirm that the wavelength of the maximum excitatory response to visible light is ~560 nm (Fig. 2C, Additional file 1: Figure S3), which is consistent with the previous report [4]. The wavelength of the maximum response is almost consistent with that of absorption maxima of parietopsin with A2 retinal (~570 nm, Fig. 2A), supporting the idea that parietopsin is responsible for visible light sensitivity in the chromatic response of the lamprey pineal organ.

### Characterization of parietopsin-expressing photoreceptor cells

We investigated several properties of the parietopsin-expressing photoreceptor cells, namely the G protein colocalized with parietopsin and a single-cell response. In the side-blotched lizard parietal eye, parietopsin is coupled with Go-type G protein, contributing to the light-dependent depolarizing response of the photoreceptor cells [13]. We also found that the Go-alpha subunit is co-expressed with parietopsin (and also parapinopsin) in photoreceptor cells of the zebrafish pineal organ [12]. Thus, we investigated whether parietopsin is colocalized with the Go-type G protein in the lamprey pineal organ. Immunohistochemical analyses with antibody against Go-type G protein showed that Go was localized in parietopsin-expressing cells (Fig. 2D–F) but not in parapinopsin-expressing cells where Gt was localized [8]. We then investigated the light response in a single parietopsin-expressing photoreceptor cell using intracellular recording. We successfully measured the depolarizing response to green light (Fig. 2G–I, Additional file 2: Movie S1), which is opposite to a hyperpolarization to light in the parapinopsin-expressing cells of the lamprey pineal organs [11] and similar to depolarizing response to green light based on parietopsin photoreception in the photoreceptor cells of the lizard parietal eye [13, 14]. Therefore, we suggest that parietopsin is responsible for visible light sensitivity in the chromatic response of the lamprey pineal organ.

### Neural connection between photoreceptor and ganglion cells

**Fig. 3:**
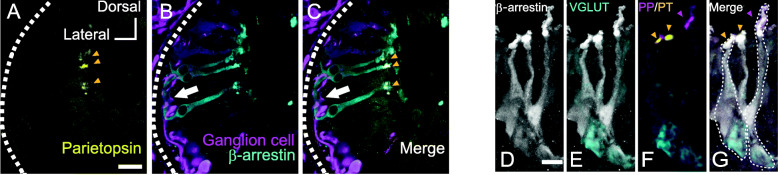
Characteristics of parietopsin-expressing photoreceptor cells in the lamprey pineal organ. **A**–**C** Fluorescence triple staining images showing parietopsin (**A**, yellow, arrowheads show outer segments), β-arrestin (**B**, cyan, whole cell), and ganglion cells labeled by retrograde tracing with neurobiotin (**B**, magenta). The white arrow shows the contact between the parietopsin photoreceptor cells and ganglion cells (**C**). The dotted trace indicates the outline of the pineal organ. **D**–**G** Fluorescence quadruple staining images showing localization of β-arrestin (**D**, white), VGLUT (**E**, cyan), parietopsin (**F**, yellow), and parapinopsin (**F**, magenta). Landmarks with broken line indicate outlines of photoreceptor cells. The yellow and magenta arrowheads in **F** and **G** show the outer segments of parietopsin- and parapinopsin-expressing photoreceptor cells, respectively. Both parapinopsin- and parietopsin-expressing photoreceptor cells employ glutamate as a neurotransmitter. Scale bar = 20 μm (**A**), 10 μm (**D**)

We examined the neural connections between parietopsin-expressing photoreceptor cells and ganglion cells to elucidate how parietopsin- and parapinopsin-expressing photoreceptor cells regulate chromatic responses in ganglion cells. We performed retrograde labeling of pineal ganglion cells from the pineal tract. We then stained pineal photoreceptor cells with antibodies against lamprey parietopsin (staining outer segments) and against β-arrestin, which stains whole parietopsin-expressing cells (Additional file 1: Figure S4) in addition to parapinopsin-expressing cells [19]. The triple staining images clearly show that the parietopsin-expressing cells are close enough to the ganglion cells to contact them directly (Fig. 3A–C). These observations together with our previous finding that parapinopsin-expressing cells directly connect to the ganglion cells in the lamprey pineal organ [19] suggest that both the parietopsin-expressing and parapinopsin-expressing cells directly transmit light information to the ganglion cells. Next, we immunohistochemically investigated the localization of vesicular glutamate transporter (VGLUT) as a marker for glutamatergic neurons in the photoreceptor cell terminal by immunostaining with the antibody against β-arrestin. Quadruple staining images showed that VGLUT was expressed in the terminal regions of photoreceptor cells, each of which expresses parapinopsin or parietopsin (Fig. 3D–G). Thus, glutamate is utilized as a neural transmitter in both parapinopsin- and parietopsin-expressing cells. The lamprey parietopsin- and parapinopsin-expressing cells depolarize and hyperpolarize to UV and green light (visible light), respectively, suggesting that light-dependent changes of the effective neurotransmitter level on the ganglion cells, which is caused by light-dependent increases and decreases of the neurotransmitter amount released from parietopsin- and parapinopsin-expressing cells, respectively. In other words, parietopsin and parapinopsin contribute to the generation of color opponency in the pineal ganglion cells with two types of photoreceptor cells, which is referred to as “two-cell system.”

## Discussion

In this paper, we found that the parietopsin gene is present in the river lamprey and expresses in the pineal and parapineal organs. In situ hybridization analysis revealed that parietopsin is more highly expressed in the parapineal organ than in the pineal organ. The high expression level in the parapineal organ supports the evolutionary scenario that the lizard parietal eye, in which parietopsin expression was first discovered, was derived from the parapineal organ during vertebrate evolution.

### Color opponency involving two kinds of photoreceptor cells expressing parapinopsin and parietopsin, respectively, in the lamprey pineal organ

We discovered that in the lamprey pineal organ, parietopsin is localized in photoreceptor cells distinguished from those expressing parapinopsin, and is involved in pineal wavelength discrimination, which is referred to as a “two-cell system.”

In the lamprey pineal organ, a previous study revealed that the pineal ganglion cells locate in the ventral and peripheral/surrounding regions and are highly rare in the dorsal central region [16], and the color opponency, namely antagonistic response to UV and visible lights, is observed in the ganglion cells that mainly locate in the peripheral region [4]. We found that a larger number of the parietopsin-expressing cells were localized in the periphery and its surroundings than in the dorsal central region (Fig. 1C, Additional file 1: Figure S2B and C), showing that the distribution of parietopsin-expressing cells is suitable to make their connection with the chromatic ganglion cells. In contrast, the parapinopsin-expressing photoreceptor cells are present not only in the periphery and its surroundings but also in the dorsal central region (Fig. 1B, Additional file 1: Figure S2A and C). Our previous observation suggested that light signals captured by the dorsal photoreceptor cells expressing parapinopsin were possibly transferred to peripheral ganglion cells via the extended processes of the parapinopsin-expressing cells to peripheral regions and/or via gap-junction-mediated connections among parapinopsin-expressing cells [11, 16]. Therefore, it can be speculated that the color opponency in the peripheral ganglion cells is achieved by photoreception by parietopsin-expressing cells in the peripheral regions and parapinopsin-expressing cells in the peripheral and dorsal regions.

Previous studies revealed that parietopsin is colocalized with a shorter wavelength-sensitive opsin, e.g., parapinopsin or pinopsin, to achieve color opponency in the photoreceptor cells (“one-cell system”), as observed in the zebrafish pineal organ and the lizard parietal eye [12, 13]. These findings indicate that parietopsin serves as a green-sensitive opsin involved in different cellular mechanisms to achieve color opponency in the pineal and pineal-related organs, suggesting that parietopsin is a common molecular basis for visible light sensitivity in the pineal-related wavelength discrimination in a wide variety of vertebrates from cyclostomes to reptiles.

We found that the parietopsin-expressing cells contain Go-type G protein (Go), producing a depolarizing response to light in the lamprey pineal organ (Fig. 2D–I). Since previous research revealed that parietopsin evokes depolarization to green light via Go activation in the one-cell system of the lizard parietal eye, our findings suggest that parietopsin evokes the depolarizing response via signal transduction mediated by Go in the lamprey two-cell system and the combination of parietopsin and Go may be highly conserved to generate depolarizing responses in pineal photoreceptor cells. Conversely, parapinopsin-expressing cells hyperpolarize to UV light via activation of transducin (Gt), as observed in rod and cone visual cells.

Furthermore, we found that the vesicular glutamate transporter is localized in the terminal region of both parietopsin- and parapinopsin-expressing cells in the lamprey pineal organ (Fig. 3D–G). This finding demonstrates that both types of photoreceptor cells utilize glutamate to transmit light signals to ganglion cells. The visible light-dependent depolarization of the parietopsin-expressing photoreceptor cell induces glutamate release, increasing the neural firings in ganglion cells, whereas UV-light-dependent hyperpolarization of the parapinopsin-expressing cells decreases glutamate release, suppressing the neural firings. Thus, the two photoreceptors cooperatively regulate the neural firing level. The spontaneous neural firings observed under dark conditions are considered to be generated by glutamate released from the parapinopsin-expressing cells that maintain depolarized potential in the dark. It can be speculated that the antagonistic response to UV and visible light stimuli in the ganglion cells is generated by subtraction and addition of neural firings from and to the spontaneous neural firings due to hyperpolarization-dependent decreases and depolarization-dependent increases of the glutamate released from parapinopsin-expressing photoreceptor cells and parietopsin-expressing photoreceptor cells, respectively (Fig. 2H).

### Functional comparison of the one- and two-cell systems involving the same two opsins

Our previous studies showed that the photoreceptor cells of the zebrafish pineal organ contain both parietopsin and parapinopsin as well as both Go and Gt (one-cell system) [8, 12]. Furthermore, we immunohistochemically confirmed that the photoreceptor cells of the green iguana parietal eye also contain the same molecular components (Additional file 1: Figure S5). A previous study [13] reported that two bleaching opsins (parietopsin and pinopsin [17, 20]) within single photoreceptor cells in the side-blotched lizard parietal eye contribute to the generation of color opponency via Go and gustducin (Ggust, phylogenetically classified into Gt-type G protein group [21]) activation, which enables the detection of dawn and dusk [14]. Therefore, because parietopsin is a bleaching opsin, zebrafish and iguana photoreceptor cells are considered to generate color opponency with parietopsin and parapinopsin via Go and Gt/gust under “weak” light conditions such as dawn and dusk where the number of photons is limited. In addition, our previous study suggested that zebrafish pineal photoreceptor cells can generate color opponency under “strong” light conditions with parapinopsin alone due to its bistable nature [12]. Therefore, we can speculate that under weak light conditions, parietopsin and parapinopsin cooperatively achieve color opponency in the zebrafish pineal organ and the iguana parietal eye via activation of Go and Gt/gust, respectively, similarly to the lizard system. Conversely, under strong light conditions, the bleaching property of parietopsin may result in the release of its chromophore, preventing the efficient capture of visible light. In fact, our previous calcium-imaging study on the parapinopsin and parietopsin-co-expressing cells of the zebrafish pineal organ revealed that the decreases in calcium level in response to strong white light were almost identical between WT and parietopsin-KO zebrafish [12], suggesting that the parietopsin-Go cascade negligibly affects the calcium level change evoked by parapinopsin-photoreception under strong white light. Accordingly, it is suggested that under strong light conditions such as in the late afternoon [12], color opponency is generated by regulating the activation level of the parapinopsin-Gt cascade alone, even in the one-cell system involving parapinopsin and parietopsin.

The parapinopsin-based color opponency in zebrafish is based on the formation and shift of the photoequilibrium between the dark state and photoproduct because of molecular properties of zebrafish parapinopsin, namely, the absorption characteristics and stabilities of two states. Lamprey parapinopsin has almost the same molecular properties as the zebrafish one, suggesting that the lamprey parapinopsin-expressing cells exhibit color opponency based on parapinopsin alone, under light conditions strong enough to evoke photoequilibrium between the dark state and photoproduct of parapinopsin. In fact, we confirmed that lamprey parapinopsin regulated the second messenger, cAMP level depending on light colors, e.g., UV, blue, and green lights in cultured cells (Additional file 1: Figure S6), which indicated that parapinopsin potentially generates color opponency with its bistability under strong light conditions in the lamprey pineal organ. In such light conditions, however, parietopsin-expressing cells continue to release glutamate at their nearly maximal level against ganglion cells receiving glutamate from parapinopsin-expressing cells. Therefore, the glutamate released from the parietopsin-expressing cells reduces the change ratio in glutamate from the parapinopsin-expressing cells, which results in the deterioration in signal-to-noise ratio for color opponency based on parapinopsin alone in the ganglion cells. Taken together, even if the parapinopsin-based color opponency is generated in the parapinopsin-expressing cells of the lamprey two-cell system, the lamprey system may not be tuned for strong light conditions, unlike the zebrafish one-cell system.

### A proposed evolutionary scenario for the molecular and cellular bases of pineal-related color opponency

As discussed above, under weak light conditions, parapinopsin and parietopsin achieve color opponency in both one-cell system and two-cell systems, whereas, under strong light conditions, parapinopsin-based color opponency in the photoreceptor cell transmits to the ganglion cell with high signal-to-noise ratio in the one-cell system but not the two-cell system. Therefore, color opponency in the ganglion cells of one-cell systems is considered superior to that of two-cell systems for variations of natural light intensity. In the viewpoint of the costs associated with neurons, it can be also speculated that the one-cell system is superior to the two-cell system. “Neurons,” including photoreceptor cells, consume energy to the necessity to maintain membrane potential and support neurotransmission, suggesting that the one-cell system could lead to the cost-cutting for pineal color opponency rather than the two-cell system.

**Fig. 4:**
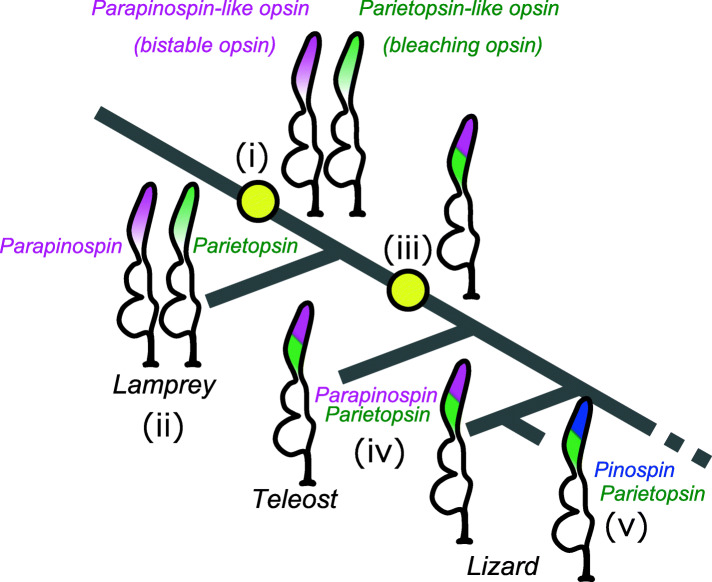
The proposed evolutionary model for color opponency systems in vertebrate pineal-related organs. Bold lines indicate branches of the vertebrate lineage. Speculated ancestral photoreceptor cell types and current types for the pineal color discrimination are drawn on each key node. Please see the “Discussion” section for the detailed descriptions of points (i)–(v)

Each of one-cell and two-cell systems is considered to have a similar possibility for the ancestral system from the viewpoint of the phylogenetic relationship of agnathostome (two-cell system) and gnathostome (one-cell system). It is the general concept that color vision involving multiple photoreceptor cells emerged from a monochromatic vision involving a single kind of photoreceptor cell. However, in the case of the evolution in the pineal color opponency from the ancestral to present-day vertebrates, such an evolutionary direction, from the one-cell to the two-cell system, would be unlikely because it leads the system to decrease the signal-to-noise ratio in generating color opponency in the ganglion cells, with high cost to separate the distribution of functional molecules involved in the system. From these points of view, we propose a more plausible scenario as follows (Fig. 4): (i) In the common ancestor of vertebrates, the two-cell system involving photoreceptor cells expressing parapinopsin-like UV-sensitive bistable opsin and those expressing parietopsin-like green-sensitive bleaching opsin cooperatively generated color opponency in the pineal organ. (ii) The two-cell system was maintained in the agnathostome lineage as found in the present-day lamprey. (iii) After the gnathostome-agnathostome split, the one-cell system emerged from the two-cell system by integrating two opsins and two G proteins into a single kind of photoreceptor cell in the gnathostome lineage. The co-expression of two opsins and two G proteins in a single cell (one-cell system) enabled the ganglion cell to achieve color opponency suitable for strong light conditions with high signal-to-noise ratio under strong light conditions without loss of color opponency under weak light conditions. (iv) The advanced color opponency system with one-cell and two-opsin was inherited in teleost and lizard lineages. (v) In addition, in the lineage leading to the side-blotched lizard, parapinopsin was replaced with a blue-sensitive bleaching opsin, pinopsin. The replacing parapinopsin with pinopsin may increase the sensitivity to light for detection of the global color changes at dawn and dusk, because pinopsin has been reported to have the potential to be employed for the scotopic vision, like rhodopsin [22]. Simultaneously, a shift of wavelength sensitivity from UV (based on parapinopsin) to blue (based on pinopsin) might have occurred. Following this scenario, we suggest that the evolutionary direction of the color opponency systems for the pineal and related organs, “two-cell system (multiple-cell system)” to “one-cell system,” might be different from that for color vision in which “multiple-cell system” was optimized with developing complexed neural circuits in the retina for generating color opponency.

## Conclusion

In this study, we showed that the jawless vertebrate, lamprey, employs a “two-cell system” for generating color opponency in the pineal ganglion cells, which is different from the pineal “one-cell system” in known jawed vertebrates although both systems involve the same set of opsins, parietopsin and parapinopsin. The findings that the two types of cells utilize the same neurotransmitter, glutamate, suggest that in the ganglion cells of the two-cell system, under strong white light conditions, even if the parapinopsin alone generate color opponency like in a one-cell system as we previously reported [12], the change in glutamate amount released from the parapinopsin-expressing cells as the chromatic signals may be masked by a large amount of glutamate released from the parietopsin-expressing cells as a saturated signal, canceling the chromatic signals from the parapinopsin-expressing cells. In this point of view, it can be speculated that lampreys have “maintained” a two-cell system that works exclusively under limited weak light conditions. In contrast, the one-cell systems in other vertebrates might have evolved from a two-cell system to adjust to a wider range of light intensity, which implies an evolutionary direction of cellular bases distinct from that of color vision in eyes.

## Methods

### Lampreys

Lampreys (*Lethenteron camtschaticum*) were commercially obtained and maintained on 12-h light/12-h dark cycles at 4°C. All animal procedures were approved by the Osaka City University Animal Experiment Committee (#S0032) in compliance with the Regulations on Animal Experiments from Osaka City University.

### Lamprey parietopsin cDNA cloning

The total RNAs extracted from the lamprey pineal organ, retina, and brain were reverse transcribed to cDNAs using oligo (dT) primers. The cDNAs were used as templates for PCR amplification. The following sense and antisense primers were used to obtain the parietopsin cDNA fragments from lamprey: sense primer 5′-CAAGAAGATTAAGCGCGTGG-3′; antisense primer 5′-TTGGCGGTTGAGGAAGAAGT-3′. These primers were designed based on the genome sequences of marine lamprey (*Petromyzon marinus*) from the Broad Institute Genome Project (www.broadinstitute.org). The full-length lamprey parietopsin cDNA was obtained using 3′ and 5′ RACE systems (Invitrogen). The 3′ and 5′ RACE primers are listed in Additional file 1: Table S1.

### Phylogenetic tree inference

Multiple alignment of the amino acid sequences of opsins was conducted with the aid of the XCED software [23]. The molecular phylogenetic tree was inferred by the neighbor-joining method [24]. Bootstrap analysis was conducted by the method of Felsenstein [25]. Accession numbers of the sequence data from the NCBI Genbank/RefSeq database (http://www.ncbi.nlm.nih.gov/) and Ensembl version 79 (http://www.ensembl.org/) are as follows: mosquito Opn3, AB753162; annelid c-opsin, AY692353; zebrafish Opn3, EF043381; human OPN3, AF140242; river lamprey parietopsin, LC600861; pouched lamprey parietopsin, KT749684; clawed frog parietopsin, DQ284780; side-blotched lizard parietopsin, DQ100320; iguana parietopsin, AB626970; zebrafish parietopsin, KT008406; elephant shark parapinopsin, AFP03346; pouched lamprey parapinopsin 53253-2-1, ANV21070; pouched lamprey parapinopsin 53253-1-3, ANV21069; river lamprey bPPL, AB116385; river lamprey parapinopsin, AB116380; clawed frog parapinopsin, AB159672; iguana parapinopsin, AB626969; anole parapinopsin, AB626968; spotted gar parapinopsin, ENSLOCT00000017485; catfish parapinopsin, AF028014; zebrafish PP1, AB626966; pufferfish PP1, AB626964; rainbow trout PP1, AB159673; zebrafish PP2, AB626967; pufferfish PP2, AB626965; rainbow trout PP2, AB675727; Ci-opsin 1, AB058682; river lamprey P-opsin, LC500595; zebrafish valop-A, AB035277; zebrafish valop-B, AY996588; chicken VAL opsin, GQ280390; toad pinopsin, AF200433 chicken pinopsin, U15762; river lamprey red, AB116381; chicken iodopsin, X57490; goldfish red, L11867; mouse green, AF011389; human red, AH005298; human green, AH005296; goldfish UV, D85863; chicken violet, M92039; mouse UV, U92562; human blue, AH003620; goldfish blue, L11864; chicken blue, M92037; goldfish green, L11866; chicken green, M88178; river lamprey rhodopsin, AB116382; goldfish rhodopsin, L11863; chicken rhodopsin, D00702; mouse rhodopsin, M55171; and human rhodopsin, U49742.

### Double fluorescence in situ hybridization

Preparation of the RNA probes and in situ hybridization were carried out as previously described [8]. Digoxigenin (DIG)- and fluorescein-labeled antisense and sense RNA probes for lamprey parapinopsin and parietopsin mRNAs were synthesized using the DIG RNA-labeling kit and fluorescein RNA-labeling kit (Roche), respectively. Sections were pre-treated with proteinase K and hybridized with each RNA probe in ULTRAhyb Ultrasensitive Hybridization Buffer (Ambion). The pineal sections hybridized with DIG-labeled probes were incubated with horseradish peroxidase (HRP)-conjugated anti-DIG antibody (Roche) and subsequently treated with the TSA plus DNP (HRP) system (Perkin Elmer), followed by incubation with Alexa 488-conjugated anti-DNP antibody. The fluorescein-labeled probes were detected through incubation with alkaline phosphatase-conjugated anti-fluorescein antibody (Roche) followed by a color reaction using the HNPP Fluorescent Detection Set (Roche).

### Antibodies

The antibodies against the lamprey parietopsin and parapinopsin were generated against the C-terminal 55-, 50-amino-acid region of the proteins, respectively. The anti-β-arrestin antibody was generated against 269 amino acids (V29–E297) of the protein. The antigens were prepared using the pMAL Protein Fusion and Purification System (New England Biolabs), following the previously reported methods [10], and immunized to the chicken (anti-parietopsin antibody), mouse (anti-parapinopsin antibody), and guinea pig (anti-β-arrestin antibody). Antiserums obtained from these immunized animals were used for immunohistochemistry. The anti-VGLUT (Synaptic Systems, #135 503), anti-Go-type G protein (MBL, #551), and anti-Gt/gust-type G protein (CytoSignal, #TF15) antibodies were commercially obtained. Antibodies against iguana parapinopsin and parietopsin were previously generated [10].

### Immunohistochemistry and retrograde labeling

The dissected lamprey pineal organs and green iguana parietal eyes were fixed in 4% paraformaldehyde, cryoprotected in 0.1 M phosphate buffer containing 30% sucrose, frozen with OCT Compound (Sakura Finetechnical), and sectioned at 20 μm. For quadruple staining of lamprey pineal sections, antisera containing the anti-lamprey parietopsin, anti-parapinopsin, and anti-β-arrestin antibodies were diluted 1:500 with PBS containing 0.1% tween, 0.5% triton-X, and 10% normal goat serum. Pineal sections were incubated with the diluted antisera, except the anti-VGLUT antibody, at 4°C overnight, followed by incubation with Alexa Fluor 488 anti-chicken IgG, Alexa Fluor 405 anti-mouse IgG, and Alexa Fluor 647 anti-guinea pig IgG (Thermo Fisher Scientific) at room temperature. Subsequently, the sections were incubated with 1:200 diluted anti-VGLUT in PBS at 37°C overnight, followed by incubation with Alexa Fluor 594 anti-rabbit IgG at room temperature. The antibodies used for immunostaining of iguana parietal eye sections are the anti-iguana parietopsin (1:500, mouse antiserum), anti-iguana parapinopsin (1:500, rabbit antiserum), anti-Gt/gust-type G protein (1:500, mouse IgG), and Go-type G protein (1:200, rabbit IgG) antibodies. The parietal eye sections were incubated with the antibody dilution at 4°C overnight, followed by incubation with Alexa Fluor 488 anti-mouse IgG and Alexa Fluor 594 anti-rabbit IgG (Thermo Fisher Scientific) at room temperature. Neurobiotin (Vector laboratories) was used for retrograde labeling. In brief, the neurobiotin powder was loaded onto the lamprey pineal tract after the decapitation and surgical treatment of the pineal organ with forceps. After 30 min, the pineal organ was washed with PBS and incubated at 4°C for 16 h, followed by fixation with 4% paraformaldehyde. The immunohistochemistry procedure after fixation was performed as described above. The neurobiotin was labeled with Alexa Fluor 594 streptavidin (Thermo Fisher Scientific). All fluorescence images were captured using confocal laser scanning microscopes (Leica, TCS SP8).

### Electrophysiological intracellular and extracellular recording

Before electrophysiological recording, lampreys were kept on ice in darkness for at least 30 min before decapitation. The pineal organ and brain were dissected and immersed in the ringer solution (122.4 mM NaC1, 2.7 mM KC1, 1.8 mM CaC12, and 4 mM HEPES; pH 7.5). Electrodes for intracellular recordings were made from glass capillaries (BF100-58-10, Sutter Instrument) with a Sutter puller (Model P-97, Sutter Instrument). The resistance of electrodes containing 2% neurobiotin in 3M KCl ranged from 100 to 150 MΩ. Intracellular responses were amplified by IR-183 (Cygnus Technology, Delaware Water Gap, PA). Carbon fiber electrodes (Carbostar-1, Kation Scientific #E011-20) were used for extracellular recording. Neural firings of the pineal ganglion cells were amplified by ER-1 (Cygnus Technology, Delaware Water Gap, PA). Data acquisitions of all recordings were performed with PowerLab (ADI Instruments). The monochromatic light for stimuli was equalized (∼1.0 × 10^13^ photons per cm^2^ s at Log I = 0) and delivered through interference filters (half bandwidth, 20 nm) and neutral density filters from a 500-W Xenon arc lamp. After intracellular recordings, the electrophoretic neurobiotin injections were administered. The neurobiotin-loaded pineal organs were visualized with Alexa Fluor 594 streptavidin (Thermo Fisher Scientific) following fixation and sectioning as described above. In the analyses of outputs from extracellular recordings, subtracted values of the action potential numbers for 4 s before and during light stimuli (Log I = −0.7) were calculated. The values were normalized with the maximum mean values (at ~560 nm). The relative mean and standard error values were obtained based on 6 repeated extracellular recordings.

### Spectroscopy

The cDNA of lamprey parietopsin was tagged with the monoclonal antibody Rho 1D4 epitope sequence (ETSQVAPA). The tagged parietopsin cDNAs were inserted into a pcDNA3.1 (Invitrogen). Pigment expression in HEK293S cells and purification were carried out as described previously [26]. Briefly, to reconstitute the pigment, the expressed proteins were incubated overnight with excess 11*-cis* retinal or 11*-cis* 3-dehydororetinal. The pigments were then extracted using 1% dodecyl β-D-maltoside (DM) in 50 mM HEPES buffer (pH 6.5) containing 140 mM NaCl (buffer A). To purify the pigment, pigments in the crude extract were bound to 1D4-agarose, washed with 0.02% DM in buffer A (buffer B), and eluted with buffer B containing the 1D4 peptide. The spectra of purified parietopsin reconstituted with *11-cis* retinal and *11-cis* 3-dehydroretinal were recorded with 100 mM hydroxyl amine. The pigment absorption spectra were recorded at 0°C with a Shimadzu UV2450 spectrophotometer. Green light was generated using a 1-kW halogen lamp (Philips) equipped with a 550-nm interference filter, and an O53 glass cutoff filter (Toshiba), respectively.

### Measurement of intracellular cAMP level

The intracellular cAMP level changes in lamprey parapinopsin-expressing HEK293S cells were measured using the GloSensor cAMP assay (Promega) as previously described [27]. HEK293S cells (20–30% confluent) in 35-mm tissue culture dishes were transfected with 1.5 μg each of the lamprey parapinopsin and the pGloSensor-22F cAMP (Promega) plasmid using the PEI transfection method, followed by incubation overnight in a culture medium containing 10% FBS. Following the overnight incubation after the supplement of 11-cis retinal to the medium, the medium was replaced with a CO2-independent medium containing 10% FBS and GloSensor cAMP Reagent stock solution (Promega). Luminescence, representing the amount of cAMP, was measured at 25°C using a GloMax 20/20n Luminometer (Promega).

## Supplementary Information

**Additional file1: Figure S1-S6 and Table S1. Figure S1. Phylogenetic position of lamprey parietopsin.** The tree was inferred by the neighbor-joining method using the Opn3 homologs as an outgroup. The lamprey opsin isolated in the current study is classified into parietopsin (PT) group. Abbreviations indicating subgroups of opsins are as follows: PT, parietopsin; PPL, parapinopsin-like; PP, parapinopsin; VA/VAL, VA- and VAL-opsin; P, pinopsin; LWS, long wavelength-sensitive opsin; SWS1, short wavelength-sensitive type1 opsin; SWS2, short wavelength-sensitive type2 opsin; MWS, middle wavelength-sensitive opsin; RH, rhodopsin. The position of lamprey parietopsin is shown with bold character. Bootstrap probabilities (≥80%) are indicated at each branch node. The scale bar indicates 0.1 substitutions per site. **Figure S2. The distribution patterns of parapinopsin and parietopsin in the lamprey organ (Related to Figure** **1****).** (A, B) Images by *in situ* hybridization analyses, showing expression patterns of parapinopsin (A), and parietopsin (B). (C) The double stained image with the antibodies against parapinopsin (magenta) and parietopsin (green), indicating biased expression of parietopsin in the peripheral and its surroundings region rather than in the dorsal central region of the pineal organ. (D)The schematic presentation showing the expression patterns of parapinopsin (magenta) and parietopsin (green) in the lamprey pineal organ. The dotted line indicates the location of the pineal slice used in Fig. 2G. Scale bars = 100 μm. **Figure S3. Details of extracellular recording analyses (Related to Figure** **2****B and C).** The relative response profiles of the visible light-sensitive component in pineal chromatic responses. The six color traces (open circles) were obtained with six-repeated set of light stimuli (460-640 nm). The black closed circles represent the mean values obtained from the six traces and are the same values as shown in Fig. 2C. **Figure S4. Parietopsin-expressing cells contain β-arrestin.** (A-C) Fluorescence double staining images by immunohistochemical analyses showing expression patterns of parietopsin (A, green), β-arrestin (B, magenta). Merged image (C) shows parietopsin-expressing cells contain β-arrestin. Arrowheads show the outer segments of parietopsin-expressing cells. Scale bar = 20 μm. **Figure S5 Co-expression of two opsins and two G proteins in the iguana parietal eye photoreceptor cells.** (A-C) Fluorescence double staining images by immunohistochemical analyses showing the co-expression of molecular components in photoreceptor cells of iguana parietal eyes. The images show (A) the co-expression of Go-type (Go, green) and Gt/gust-type (Gt/gust, magenta), (B) the co-expression of Gt/gust-type G protein (green) and parapinopsin (PP, magenta), and (C) the co-expression of parietopsin (PT, green) and Go-type G protein (magenta) in the iguana parietal eye photoreceptor cells. OS indicates the layer of outer segments of the photoreceptor cells. Scale bar = 20 μm. **Figure S6. Changes in cAMP levels of parapinopsin-expressing cultured cells depending on color of light.** Changes in cAMP levels in lamprey parapinopsin-expressing HEK293S cells using GloSensor. The adenylyl cyclase activator forskolin (3.5 μM) was added to the culture medium (35-mm dish, ~1.2 × 10^6^ HEK cells) to elevate the intracellular cAMP level (black arrow). The arrowheads show ~400-nm (magenta, ~2.5 × 10^15^ photon/cm^2^/s), ~455-nm (blue, ~4.9 × 10^16^ photon/cm^2^/s), and ~505-nm (green, ~2.6 × 10^16^ photon/cm^2^/s) monochromatic light stimuli (duration, 5s). The profile of cAMP changes clearly shows color-dependent-manner responses evoked with photoreceptions involving lamprey parapinopsin dark state and photoproduct. **Table S1. Primer list for 3’ and 5’ RACE (PDF 733 kb)**

**Additional file 2: Movie S1. The sequential images showing that the recorded photoreceptor cell is parietopsin-positive (Related to Figure****2****G and H).** Magenta indicates the dye injected into the recorded cell. Yellow indicates the outer segment expressing parietopsin. White arrowhead shows the boundary between the cell body and the outer segment. Scale bar = 10 μm. Note that the dye was loaded into the cell body but not into the parietopsin-containing outer segment because the outer segment consists of stacked membranes and limited cytosolic portion, like the case of other photoreceptor cells.

## Data Availability

All data generated or analyzed during this study are included in this published article and its supplementary information files. The sequence reported in this paper has been deposited in the DDBJ database (https://www.ddbj.nig.ac.jp/; accession no. LC600861).
