# Supplementary material for: Insights into the evolutionary origin of the pineal color discrimination mechanism from the river lamprey
Source: BMC Biol. 2021 Sep 16;19:188. doi: 10.1186/s12915-021-01121-1 (PMC8444496; doi:10.1186/s12915-021-01121-1)
Supplement: Supplementary file 1 — Additional file1: Figure S1-S6 and Table S1. Figure S1. Phylogenetic position of lamprey parietopsin. The tree was inferred by the neighbor-joining method using the Opn3 homologs as an outgroup. The lamprey opsin isolated in the current study is classified into parietopsin (PT) group. Abbreviations indicating subgroups of opsins are as follows: PT, parietopsin; PPL, parapinopsin-like; PP, parapinopsin; VA/VAL, VA- and VAL-opsin; P, pinopsin; LWS, long wavelength-sensitive opsin; SWS1, short wavelength-sensitive type1 opsin; SWS2, short wavelength-sensitive type2 opsin; MWS, middle wavelength-sensitive opsin; RH, rhodopsin. The position of lamprey parietopsin is shown with bold character. Bootstrap probabilities (≥80%) are indicated at each branch node. The scale bar indicates 0.1 substitutions per site. Figure S2. The distribution patterns of parapinopsin and parietopsin in the lamprey organ (Related to Figure 1). (A, B) Images by in situ hybridization analyses, showing expression patterns of parapinopsin (A), and parietopsin (B). (C) The double stained image with the antibodies against parapinopsin (magenta) and parietopsin (green), indicating biased expression of parietopsin in the peripheral and its surroundings region rather than in the dorsal central region of the pineal organ. (D)The schematic presentation showing the expression patterns of parapinopsin (magenta) and parietopsin (green) in the lamprey pineal organ. The dotted line indicates the location of the pineal slice used in Fig. 2G. Scale bars = 100 μm. Figure S3. Details of extracellular recording analyses (Related to Figure 2B and C). The relative response profiles of the visible light-sensitive component in pineal chromatic responses. The six color traces (open circles) were obtained with six-repeated set of light stimuli (460-640 nm). The black closed circles represent the mean values obtained from the six traces and are the same values as shown in Fig. 2C. Figure S4. Parietopsin-expressing cells co [file 12915_2021_1121_MOESM1_ESM.pdf]

Insights into the evolutionary origin of the pineal color discrimination mechanism from the river lamprey.

Authors

Seiji Wada[1,2], Emi Kawano-Yamashita[1,3], Tomohiro Sugihara[1], Satoshi Tamotsu[3], Mitsumasa Koyanagi[1,2], and Akihisa Terakita[1,2]*

Affiliations

[1]Department of Biology and Geosciences, Graduate school of Science, Osaka City University, Osaka 558-8585, Japan

[2]The OCU Advanced Research Institute for Natural Science and Technology, Osaka City University, Osaka 558-8585, Japan

[3]Department of Chemistry, Biology and Environmental Science, Faculty of Science, Nara Women's University, Nara 630-8506, Japan

* **Corresponding author:**

Akihisa Terakita

Graduate School of Science, Osaka City University

(Tel) +81-6-6605-3144, (Fax) +81-6-6605-3171

Email: terakita@osaka-cu.ac.jp

Additional file1: Figure S1-S6, Table S1

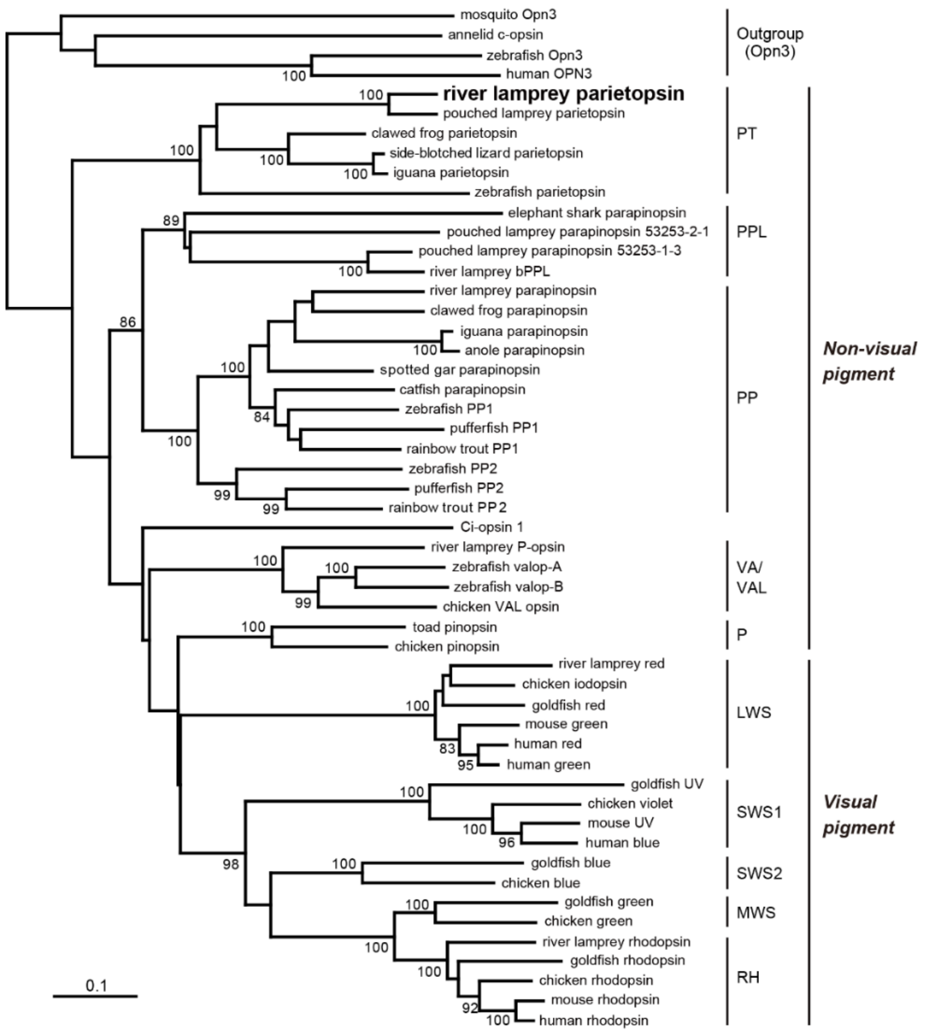

Figure S1. Phylogenetic position of lamprey parietopsin.

The tree was inferred by the neighbor-joining method using the Opn3 homologs as an outgroup. The lamprey opsin isolated in the current study is classified into parietopsin (PT) group. Abbreviations indicating subgroups of opsins are as follows: PT, parietopsin; PPL, parapinopsin-like; PP, parapinopsin; VA/VAL, VA- and VAL-opsin; P, pinopsin; LWS, long wavelength-sensitive opsin; SWS1, short wavelength-sensitive type1 opsin; SWS2, short wavelength-sensitive type2 opsin; MWS, middle wavelength-sensitive opsin; RH, rhodopsin. The position of lamprey parietopsin is shown with bold character. Bootstrap probabilities (≥80%) are indicated at each branch node. The scale bar indicates 0.1 substitutions per site.

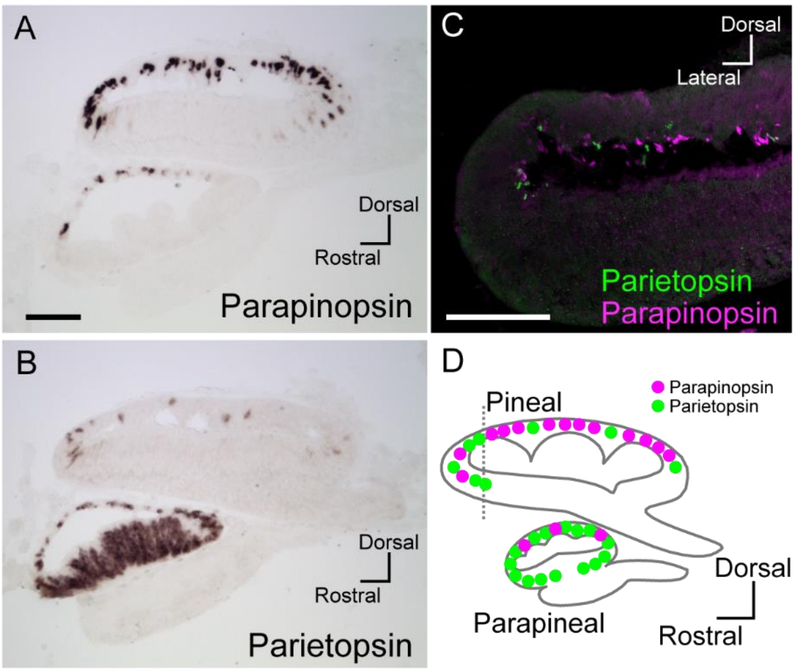

Figure S2. The distribution patterns of parapinopsin and parietopsin in the lamprey organ (Related to Figure 1).

(A, B) Images by *in situ* hybridization analyses, showing expression patterns of parapinopsin (A), and parietopsin (B). (C) The double stained image with the antibodies against parapinopsin (magenta) and parietopsin (green), indicating biased expression of parietopsin in the peripheral and its surroundings region rather than in the dorsal central region of the pineal organ. (D)The schematic presentation showing the expression patterns of parapinopsin (magenta) and parietopsin (green) in the lamprey pineal organ. The dotted line indicates the location of the pineal slice used in Fig. 2G. Scale bars = 100 μm.

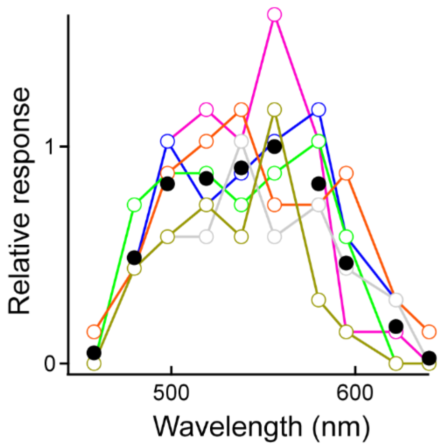

Figure S3. Details of extracellular recording analyses (Related to Figure 2B and C).

The relative response profiles of the visible light-sensitive component in pineal chromatic responses. The six color traces (open circles) were obtained with six-repeated set of light stimuli (460-640 nm). The black closed circles represent the mean values obtained from the six traces and are the same values as shown in Fig. 2C.

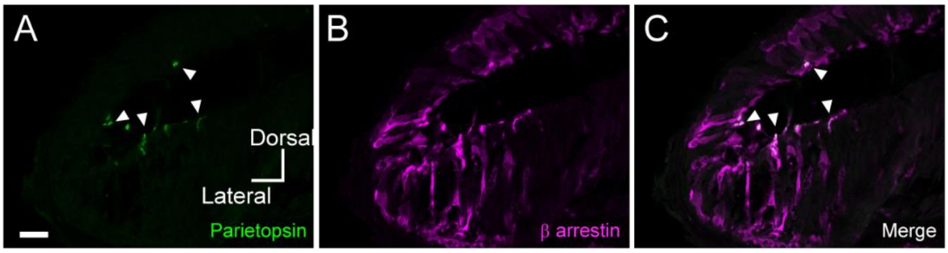

Figure S4. Parietopsin-expressing cells contain β-arrestin.
(A-C) Fluorescence double staining images by immunohistochemical analyses showing expression patterns of parietopsin (A, green), β-arrestin (B, magenta). Merged image (C) shows parietopsin-expressing cells contain β-arrestin. Arrowheads show the outer segments of parietopsin-expressing cells. Scale bar = 20 μm.

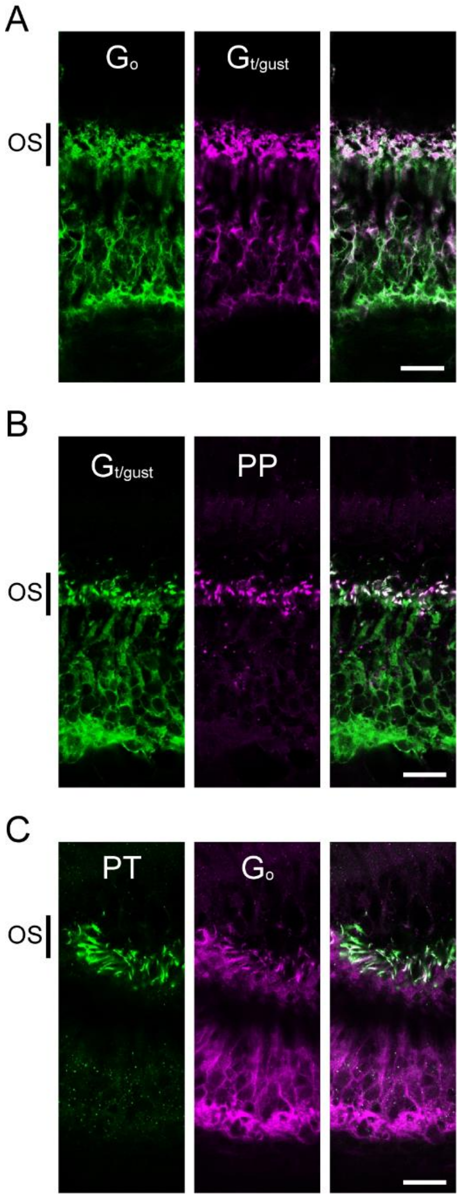

Figure S5. Co-expression of two opsins and two G proteins in the iguana parietal eye photoreceptor cells.

(A-C) Fluorescence double staining images by immunohistochemical analyses showing the co-expression of molecular components in photoreceptor cells of iguana parietal eyes. The images show (A) the co-expression of Go-type (Go, green) and Gt/gust-type (Gt/gust, magenta), (B) the co-expression of Gt/gust-type G protein (green) and parapinopsin (PP, magenta), and (C) the co-expression of parietopsin (PT, green) and Go-type G protein (magenta) in the iguana parietal eye photoreceptor cells. OS indicates the layer of outer segments of the photoreceptor cells. Scale bar = 20 μm.

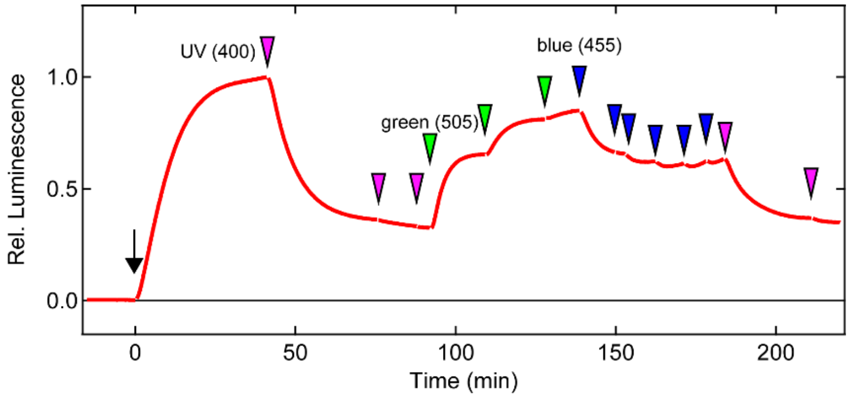

Figure S6. Changes in cAMP levels of parapinopsin-expressing cultured cells depending on color of light.

Changes in cAMP levels in lamprey parapinopsin-expressing HEK293S cells using GloSensor. The adenylyl cyclase activator forskolin (3.5 μM) was added to the culture medium (35-mm dish, ~1.2×10^6 HEK cells) to elevate the intracellular cAMP level (black arrow). The arrowheads show ~400-nm (magenta, ~2.5×10^{15} photon/cm^2/s), ~455-nm (blue, ~4.9×10^{16} photon/cm^2/s), and ~505-nm (green, ~2.6×10^{16} photon/cm^2/s) monochromatic light stimuli (duration, 5s). The profile of cAMP changes clearly shows color-dependent-manner responses evoked with photoreceptions involving lamprey parapinopsin dark state and photoproduct.

Table S1. Primer list for 3' and 5' RACE

Primers for 3'RACE	Sequence (5' - 3')
Lmprt_fw_3R0.5_Eco	ggtgaattcaactacacgtacctggtttcg
Lmprt_fw_3R1	ttaagcgcgtgggcggccac
Lmprt_fw_3R1.5	acccgcgggaggagatgcgt
Lmprt_fw_3R2	gggcgcgttcatggcgtgct
Lmprt_fw_3R2.5	ctgcccatgtacttcgccaa
Lmprt_fw_3R3	tctacttcttcctcaaccgc
**Primers for 5'RACE**	
Lmprt_rv_5R1	gcggttgaggaagaagtaga
Lmprt_rv_5R1.5	ttggcgaagtacatgggcag
Lmprt_rv_5R2	agcacgccatgaacgcgccc
Lmprt_rv_5R2.5	acgcatctcctcccgcgggt
Lmprt_rv_5R3	gtggccgcccacgcgcttaa
Lmprt_rv_5R3.5	ggaagtacgaaaccaggtac
Lmprt_rv_5R4	acgtttccgtatgagaagac
Lmprt_rv_5R5	agaaggtccacacgaaggcc
Lmprt_rv_5R5.5	tcgagcagccgtgggtcacc
Lmprt_rv_5R6	cagcagagtgagggaccaga
